# Risk factor analysis and development of a nomogram prediction model for Plasma Cell Mastitis

**DOI:** 10.1371/journal.pone.0338711

**Published:** 2025-12-09

**Authors:** Yiming Sun, Feng Zhang, Xiaowen Ma, Wenhui Wang, Ruonan Xu

**Affiliations:** 1 Department of Neurosurgery, Tongde Hospital of Zhejiang Province, Hangzhou, Zhejiang, China; 2 Department of Breast Surgery, Hangzhou Women’s Hospital, Hangzhou, Zhejiang, China; 3 Department of Pathology, Hangzhou Women’s Hospital, Hangzhou, Zhejiang, China; 4 Department of Clinical Lab, Jinan Zhangqiu District People’s Hospital, Jinan, Shandong, China; University of South Carolina, UNITED STATES OF AMERICA

## Abstract

**Background and objective:**

The risk factors for plasma cell mastitis (PCM) remain unclear. Understanding and mitigating these factors to prevent PCM before its onset has become a significant concern. This study identifies PCM risk factors, develops a predictive nomogram, and offers insights for targeted prevention and awareness in high-risk groups.

**Methods:**

We retrospectively analyzed the clinical data of 82 patients diagnosed with PCM at Hangzhou Women’s Hospital’s Breast Surgery Department from 01/01/2019 to 01/01/2022. A control group was randomly selected, consisting of 82 healthy women aged between 20–60 years who had undergone routine health check-ups during the same period. Using SPSS 26.0 software for univariate analysis, significant risk factors for PCM were identified. R software was used for multivariate logistic regression analysis, and a nomogram prediction model for the risk of developing PCM was established.

**Results:**

The average age of patients in the study group was 32.37 ± 6.64 years, the control group was 29.54 ± 5.33 years, with no statistically significant difference between the groups (P = 0.176). The onset time after childbirth or miscarriage was 3.37 ± 1.91 years. Univariate analysis revealed significant differences in BMI, nipple retraction, number of pregnancies, recent trauma history, and hyperlipidemia (P < 0.05). Multivariate logistic regression analysis identified nipple retraction (OR=20.128, P = 0.000, 95% CI: 5.952–68.072), number of pregnancies (OR=0.343, P = 0.000, 95% CI: 0.189–0.624), and recent trauma history (within two weeks) (OR=11.154, P = 0.000, 95% CI: 2.936–42.382) as independent risk factors for PCM.

**Conclusion:**

Nipple retraction, recent trauma history, and the number of pregnancies were identified as independent risk factors for PCM. Targeted education for high-risk groups, particularly women within 3 years postpartum/post-abortion, improves disease prevention. The nomogram model had a C-index of 0.809, indicating strong discriminatory power and high prediction accuracy.

## Introduction

PCM also known as periductal mastitis, is a chronic non-lactating inflammatory disease characterized by plasma cell infiltration [1]. It predominantly affects young to middle-aged women between 20–50 years old and is known for its long disease course and tendency to recur [2]. Histologically, PCM typically shows over 50% plasma cells with varying amounts of lymphocytes, neutrophils, and eosinophils [1,3]. The disease presents as non-cyclical breast pain, nipple discharge (serous or bloody), nipple inversion, masses in the areola, non-lactating breast abscesses, and fistulas [4]. PCM must be distinguished from breast cancer (particularly inflammatory or ductal carcinoma, which exhibits irregular margins and malignant biopsy findings), acute bacterial mastitis/abscess (typically infectious, lactation-associated, and responsive to antibiotics) and granulomatous mastitis (characterized by non-caseating granulomas rather than plasma cell infiltration). Accurate differentiation relies on clinical history, imaging, and histopathology to exclude malignancy and confirm PCM’s benign inflammatory nature. The incidence of plasma cell mastitis is increasing year by year [5]. The etiology is often associated with ductal secretion and fat necrosis, leading to a massive infiltration of immune cells, including plasma cells and eosinophils [6].

Treatment options for PCM include surgery, corticosteroids, antibiotics, and traditional Chinese medicine. However, no standardized surgical methods or effective medications are available, and the clinical treatment remains challenging, with high recurrence rates and significant impacts on the aesthetic appearance of the breast [4]. The risk factors for PCM remain incompletely understood. Nipple inversion [7], elevated prolactin levels [8], and autoimmune dysfunction [9] have been identified as risk factors. Additionally, smoking, obesity, surgical trauma, abnormal ductal secretion, and degenerative ductal changes have all been proposed as possible contributing factors [1,10]. There is currently no established predictive model for assessing PCM risk in routine clinical practice.

This retrospective study analyzed 82 cases of PCM in the breast surgery department of Hangzhou Women’s Hospital, aiming to explore the associated risk factors and develop a nomogram prediction model. This model serves as an individualized risk assessment tool for PCM, enabling the prediction of disease onset in patients with relevant risk factors [8]. Beyond offering a novel perspective on PCM prevention, this study also provides valuable insights for public education and awareness, contributing to more effective disease prevention strategies.

## Materials and methods

### Clinical data

This study is a case-control study. We retrospectively analyzed the clinical features of 82 patients with PCM (study group) admitted to the Breast Surgery Department of Hangzhou Women’s Hospital from 01/01/2019 to 01/01/2022. For external validation, an independent cohort was collected from 01/02/2022 to 12/31/2023, including 37 PCM patients treated at our hospital. All cases were diagnosed pathologically (Excisional biopsy with histopathological examination or percutaneous core needle biopsy with histopathological examination).

Clinical Diagnostic Criteria for PCM: 1. Typical Clinical Presentation 1) Subareolar breast mass, often with chronic, non-lactational inflammation. 2) Associated nipple retraction, occasional abscess formation, or fistula. 3) Symptoms may include breast pain, redness, swelling, and intermittent purulent or bloody nipple discharge [11,12]. 2. Imaging Findings: 1) Mammography or ultrasound can reveal ill-defined lesions, often near the subareolar region. 2) Imaging may show ductal ectasia, thickening of duct walls, and possible microcalcifications [13]. 3. Exclusion of Other Diseases: 1) Rule out common causes of mastitis (e.g., bacterial infection) and inflammatory breast cancer. 2) Clinical diagnosis typically requires correlation of signs, symptoms, and imaging, along with patient history [1,14].

Pathological diagnostic criteria for PCM: 1. Histopathological findings: 1) Prominent infiltration of plasma cells within the duct walls and periductal tissues. 2) Chronic inflammatory changes with lymphocytes, histiocytes, and occasionally granulomatous features. 3) Possible duct ectasia and fibrosis around the affected ducts [12,14]. 2. Additional microscopic features: 1) Thickened ductal basement membrane. 2) Exudative changes or debris within duct lumens. 3) Variable degrees of granuloma formation, though not always present [15]. 4) Other histopathological features such as fat necrosis were excluded. Fat necrosis is characterized by necrotic adipocytes with disrupted cell membranes and loss of nuclear staining, surrounded by infiltrates of foamy macrophages (lipid-laden histiocytes), multinucleated giant cells, and chronic inflammatory cells [16].

Immunohistochemistry (If performed): 1) Confirmation of abundant plasma cells (positive staining for CD138). 2) Exclusion of other pathologies such as malignancy (e.g., breast carcinoma) [17].

**Fig 1:**
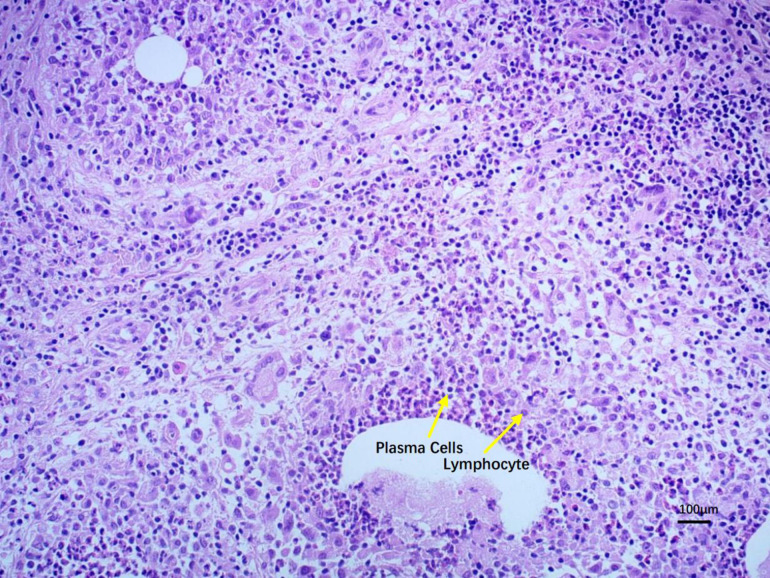
A 100x magnification image of the lesion tissue.

**Fig 2:**
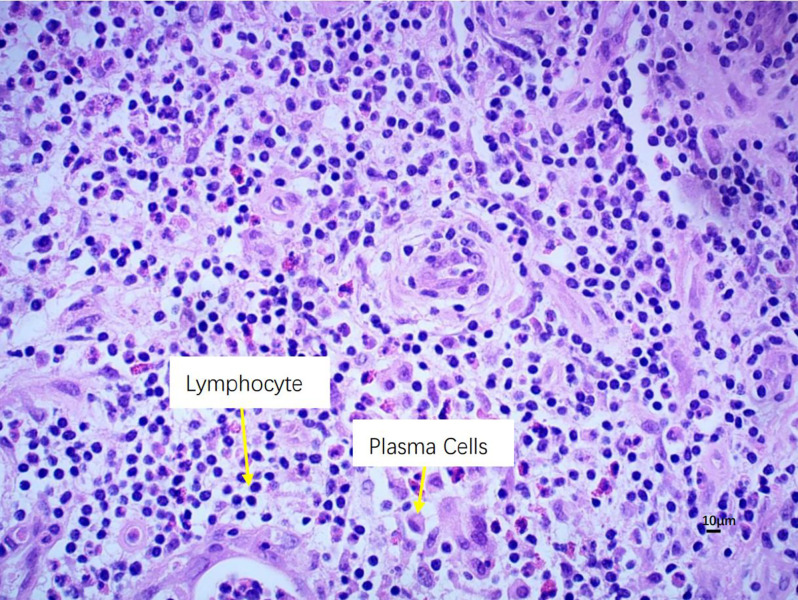
A 400x magnification image of the lesion tissue.

Indications for biopsy or surgical pathological diagnosis: 1. Atypical or unclear presentation: 1) When clinical findings and imaging studies are inconclusive. 2) If there is suspicion of a coexisting malignancy, especially inflammatory breast cancer [11]. 2. Persistent or recurrent lesions: 1) Chronic lesions that fail to respond to conservative treatment (e.g., antibiotics, observation). 2) Recurrence after initial resolution, raising concern for other pathologies [13,17]. 2. Abscess formation or fistula: 1) Abscesses not resolving with conservative management (e.g., repeated aspiration, antibiotics). 2) Fistula requiring surgical intervention for definitive treatment and accurate histopathological assessment [12,14]. 3. Guidance for treatment planning: 1) In cases where surgical excision may be considered (e.g., persistent sinus tracts or non-healing lesions). 2) To confirm PCM diagnosis before implementing targeted therapy (e.g., steroids or immunomodulatory treatments) [17] (Figs 1 and 2).

Figure shows dense infiltration of chronic inflammatory cells, including lymphocytes and plasma cells, around the ducts or acini, with a seemingly considerable proportion of plasma cells. 1. Lymphocytes: Small nuclear volume with dense, deeply stained chromatin. Minimal cytoplasm, often barely visible. Tend to infiltrate in large clusters in chronic inflammation. 2. Plasma Cells: Slightly larger than lymphocytes. Nucleus is often eccentrically located, with chromatin arranged in a “clock-face” or “cartwheel” pattern. A perinuclear halo (Golgi zone) appears as a lightly stained ring around the nucleus, creating a pale area adjacent to the nucleus in the cytoplasm. In the figure, plasma cells are generally larger than lymphocytes, with eccentrically placed nuclei and a distinct perinuclear halo.

Inclusion criteria: 1) Han Chinese women; 2) Pathologically diagnosed as PCM; 3) Available clinical data. Exclusion criteria: (1) Missing pathological confirmation; (2) Missing other key clinical variables (age, nipple retraction, pregnancy history, BMI, breast trauma history); (3) Concurrent diagnosis of other breast diseases; (4) In lactation period.

According to studies showing that most PCM patients are middle-aged women aged 30–40 years [18], and based on the National Bureau of Statistics of China defining the middle-aged population as ages 16–59, the control group was limited to women aged <60 years. A random selection method was used to choose 82 women who underwent routine check-ups at the hospital during the same period and did not have breast-related diseases as the control group. Inclusion criteria for the control group: 1) Han Chinese women; 2) No palpable mass or nodules detected on breast examination; 3) Breast ultrasound report showing ductal dilation ≤ 3 mm, no abnormal nodules; 4) Age < 60 years.

A total of 98 PCM cases were initially screened. Patients were excluded if they met any of the following:(1) Missing pathological confirmation (n = 8); (2) Missing other key clinical variables (pregnancy history, breast trauma history n = 4); (3) Concurrent diagnosis of other breast diseases (n = 1); (4) In lactation period (n = 3). After excluding patients with missing or incomplete data, a total of 82 PCM cases were included in the final analysis. The control group was selected based on a randomized 1:1 matching principle, resulting in 82 matched controls.

### Methods

General data from the study participants were collected, including age at enrollment, body mass index (BMI), blood lipid levels (serum triglycerides, total cholesterol, high-density lipoprotein, low-density lipoprotein), number of pregnancies, presence of nipple inversion, history of breast trauma (including massage and lactation history), The time interval between breast trauma and the onset of PCM, TSH levels, estrogen levels, prolactin levels, progesterone levels, oral contraceptive use, smoking history, Anti-neutrophil cytoplasmic antibodies (ANCA) (autoantibody), breastfeeding status, duration of breastfeeding, and onset time postpartum/miscarriage.

This study has been approved by the Clinical Research Medical Ethics Committee of Hangzhou Women’s Hospital in Zhejiang Province. The final ethical review opinion number is No. 2022 (6) – 03. (S1 and S2 Files) This study complies with the Declaration of Helsinki. This retrospective study was granted a waiver of the requirement for obtaining signed informed consent forms from patients. A detailed overview of the ethical oversight and human subjects protections for this study is presented in S3 File.

Breast trauma can be categorized into two types based on the timing of the injury: 1. Recent Trauma (within 2 weeks): Clinically, patients with PCM caused by common breast trauma often have a history of breast injury within 1 week. For example, a forceful impact on the breast by a child or a pet can lead to the development of PCM. Patients typically present with a breast lump within 3 days after the injury, accompanied by moderate to severe pain, and the lump tends to increase in size. Some patients may start to develop symptoms 1 week after the injury, but almost all such patients develop symptoms within 2 weeks. Therefore, the time frame for recent trauma is defined as within 2 weeks. 2.Remote Trauma (more than 2 weeks ago): This includes a history of breast injury that occurred more than 2 weeks ago, such as violent breast massage, postpartum milk expression, and other forms of physical damage.

All patients with PCM who underwent surgery in our study unit received pathological diagnosis. The pathological diagnosis is divided into two forms: 1. Lump size not exceeding a single quadrant of the breast: When there is a single lump and the size does not exceed a single quadrant of the breast, surgical removal of the lump is primarily performed for pathological examination, considering the minimal impact on breast appearance. 2. Other cases requiring pathological diagnosis: For the remaining cases, core needle biopsy or cytological examination of the abscess fluid is chosen to confirm the diagnosis, without surgical removal of the lump.

Given the significant variations in estrogen and progesterone levels during the menstrual cycle, we considered the normal values of estradiol and progesterone at different stages of the menstrual cycle. Pre-menopausal normal estradiol levels are 48–521 pmol/L in the follicular phase, 370–1835 pmol/L in the ovulatory phase, and 272–793 pmol/L in the luteal phase; pre-menopausal progesterone normal values are 0–4.8 nmol/L in the follicular phase and 7.6–97.6 nmol/L in the luteal phase. These hormonal fluctuations can be substantial and difficult to balance between the experimental and control groups, so this study did not report specific estradiol and progesterone values but categorized them as elevated or not.

Increased blood lipid levels were defined as: According to the American Heart Association (AHA) and Endocrine Society guidelines, abnormal adult blood lipid levels are diagnosed as: serum triglycerides ≥200 mg/dL (2.3 mmol/L), total cholesterol ≥240 mg/dL (6.2 mmol/L), high-density lipoprotein <50 mg/dL (1.3 mmol/L) and low-density lipoprotein ≥160 mg/dL (4.1 mmol/L) for women.

### Statistical analysis

Statistical analysis was performed using SPSS 26.0 software. For continuous variables, group comparisons were performed using an independent sample t-test (or Levene’s test if variances were unequal). Categorical data were expressed as counts and percentages; group comparisons were made using the chi-square (χ2) test (or Fisher’s exact test if any expected cell frequency was < 5). All exhibited missing rates <5% upon assessment, For continuous variables (TSH, Prolactin, Breastfeeding Duration, Onset-Weaning Interval): Predictive Mean Matching (PMM); Binary variables (Smoking, Contraceptive Use, Estrogen/Progesterone Status): Logistic Regression Imputation. Variables with a P-value < 0.05 in the univariate analysis were considered statistically significant and were further entered into a binary logistic regression model (with categorical variables converted into dummy variables) to identify independent risk factors. Only variables with p < 0.05 in the multivariable logistic regression were retained for nomogram development and performance evaluation, to reduce overfitting and ensure model stability. The linearity assumption of continuous variables in the logit was assessed using the Box-Tidwell test. For each continuous variable, an interaction term with its natural logarithm was created and included in the logistic regression model. A p-value greater than 0.05 was considered to indicate a linear relationship with the logit. R software (download available at http://www.r-project.org) was then used to construct a nomogram model based on the identified independent risk factors, utilizing the rms, Hmisc, and ggplot2 packages. We assessed model performance using the concordance index (C-index) and ROC analysis with AUC calculation to evaluate discriminative ability. Calibration was examined via bootstrap-derived calibration curves (1,000 iterations). We further assessed the model’s calibration performance using the Hosmer–Lemeshow test implemented in SPSS. The multivariate logistic regression initially considered seven predictors in 164 subjects (82 cases/controls), exceeding the 10–15 events-per-variable(EPV) threshold. Post hoc power analysis confirmed capability to detect OR≥2.0 (α = 0.05, power = 0.8). Firth’s correction was applied to address potential overfitting from the five ultimately significant predictors, with bootstrap validation ensuring parameter stability.

For external validation, we assembled an independent temporal cohort from 01/02/2022 to 12/31/2023, including 37 pathologically confirmed PCM patients treated at Hangzhou Women’s Hospital and 74 matched controls recruited from the Health Examination Center (1:2 matching). The external dataset was collected strictly according to a pre-specified protocol identical to that of the development cohort. PCM diagnosis required histopathological confirmation (excisional biopsy or ultrasound-guided core needle biopsy). Eligibility criteria, variable definitions, measurement units, and coding rules were kept fully consistent with the development set. Clinical, laboratory, and imaging variables were extracted from the electronic medical record using a standardized case report form by trained investigators, and all records were double-checked to ensure completeness and accuracy; discrepancies were resolved by consensus. Controls had no history or clinical evidence of PCM or other inflammatory breast diseases at enrollment. This standardized and temporally independent cohort ensured comparability of predictors and provided a rigorous assessment of the model’s generalizability.

All power calculations were done with G*Power 3.1 download available at https://www.psychologie.hhu.de/arbeitsgruppen/allgemeine-psychologie-und-arbeitspsychologie/gpower.

## Results

### Univariate and multivariate analysis of risk factors for PCM

The onset time after childbirth or miscarriage was 3.37 ± 1.91 years. Univariate analysis revealed significant differences in BMI, nipple retraction, number of pregnancies, recent trauma history, and hyperlipidemia (P < 0.05). Multivariate logistic regression analysis identified nipple retraction (OR=20.128, P = 0.000, 95% CI: 5.952–68.072), number of Pregnancies (OR=0.343, P = 0.000, 95% CI: 0.189–0.624), and recent trauma history (within two weeks) (OR=11.154, P = 0.000, 95% CI: 2.936–42.382) as independent risk factors for PCM. In the external validation cohort, the nomogram yielded a C-index of 0.809, with a corresponding ROC AUC of 0.809, indicating good discriminatory performance.

The average age of the research group was (32.36 ± 6.64) years, and the average age of the control group was (29.67 ± 5.33) years, with no significant difference between the two groups (χ2 = 1.85, P = 0.176). The differences in smoking (χ2 = 0.69, P = 0.405), oral contraceptive use (χ2 = 0.69, P = 0.375), ANCA (χ2 = 0.34, P = 0.560), TSH levels (mean of 2.91 ± 1.05 μmol/L in the research group, 2.97 ± 0.89 μmol/L in the control group, t = 3.42, P = 0.066), estrogen levels (both groups had normal levels, n = 164), progesterone levels (χ2 = 1.01, P = 0.316), Long term trauma history(χ2 = 1.934, P = 0.164) and prolactin levels (mean of 6.76 ± 4.59 µg/L in the research group, 6.68 ± 4.00 µg/L in the control group, t = 0.17, P = 0.677) were all not statistically significant. All patients in the research group had a history of miscarriage and/or childbirth, with an average onset time of (3.37 ± 1.91) years after delivery/miscarriage. Univariate analysis revealed significant differences in the following factors: BMI (mean of 24.20 ± 3.33 in the research group vs. 21.41 ± 2.34 in the control group, t = 12.52, P = 0.001), nipple retraction (χ2 = 34.93, P = 0.000), number of pregnancies (average of 2.05 ± 1.04 pregnancies in the research group vs. 1.34 ± 0.53 in the control group, t = 28.69, P = 0.000), recent trauma history (within two weeks) (OR=11.154, P = 0.000, 95% CI: 2.936–42.382), and hyperlipidemia (42 cases, 51.22% in the research group vs. 8 cases, 9.76% in the control group, χ2 = 33.26, P = 0.001). These differences are shown in Table 1.

**Table 1:** Analysis of risk factors for plasma cell mastitis – results of univariate analysis.

Factor	Study Group	Control Group	*t/*χ2	*P*
Number of Cases	82	82	–	–
Average Age (years)	32.37 ± 6.64	29.54 ± 5.33	1.85	0.176
Average Number of Pregnancies	2.05 ± 1.04	1.34 ± 0.53	28.69	0.000
Average BMI (kg/m2)	24.20 ± 3.33	21.41 ± 2.34	12.52	0.001
Hyperlipidemia [Cases/(%)]	42/51.22	8/9.76	33.26	0.000
Smoking [Cases/(%)]	4/4.88	2/2.44	0.69	0.405
Oral Contraceptive Use [Cases/(%)]	2/2.44	4/4.88	0.69	0.375
Recent trauma history[Cases/(%)]	31/37.80	5/6.10	24.059	0.000
Long term trauma history[Cases/(%)]	27/32.93	19/23.17	1.934	0.164
Nipple Retraction [Cases/(%)]	40/48.78	6/7.32	34.927	0.000
Average TSH Level (umol/L)	2.91 ± 1.05	2.97 ± 0.89	3.42	0.066
Estrogen Elevation [Cases/(%)]	0	0	–	–
Average Prolactin Level (ug/L)	6.76 ± 4.59	6.68 ± 4.00	0.17	0.677
Progesterone Elevation [Cases/(%)]	1/1.23	0	1.01	0.316
ANCA Positivity [Cases/(%)]	2/2.44	1/1.23	0.34	0.560
Onset Time After Childbirth/Miscarriage (years)	3.37 ± 1.91	–	–	–
Breast feeding[Cases/(%)]	69/84.15	71/86.59	0.195	0.659
Duration of breastfeeding for the last child(month)	7.43 ± 5.13	7.52 ± 4.15	−0.134	0.894
Time interval between onset and weaning(year)	1.84 ± 1,13			

Based on the univariate analysis results presented in Table 1, the 5 factors with significant differences(P < 0.05) were selected for logistic regression analysis using R software. Although the p-value for TSH was 0.066 (<0.1), indicating borderline significance, it was also included in the binary logistic regression analysis to avoid omitting any potential independent risk factors. Additionally, the long-term trauma history factor, with a p-value of 0.164, was included in the regression analysis due to its clinical importance, despite lacking clear statistical significance. Dummy variables were created for categorical data during analysis.

The Box-Tidwell test showed that all continuous variables, including BMI (p = 0.837), TSH (p = 0.551), and number of pregnancies (p = 0.959), met the linearity assumption in the logit. Therefore, these variables were retained as continuous predictors in the final logistic regression model.

Logistic regression analysis identified recent trauma history (OR=11.154, P = 0.000, 95% CI: 2.936–42.382), nipple retraction (OR = 20.128, P = 0.000, 95% CI: 5.952–68.072), and number of pregnancies (OR = 0.343, P = 0.000, 95% CI: 0.189–0.624) as independent risk factors for PCM. The remaining factors did not exhibit statistical significance. These findings are summarized in Table 2.

**Table 2:** Analysis of risk factors for plasma cell mastitis – results of multivariate analysis.

Risk Factor	p	OR	95% Confidence Interval	
Nipple Retraction	0.000	20.128	5.952	68.072
Number of Pregnancies	0.000	0.343	0.189	0.624
BMI	0.098	0.793	0.603	1.043
Hyperlipidemia	0.101	4.644	0.743	29.029
Recent trauma history	0.000	11.154	2.936	42.382
Long term trauma history	0.626	0.760	0.252	2.293
TSH	0.591	1.145	0.699	1.878

### Establishment of a prediction model for the risk of PCM

**Fig 3:**
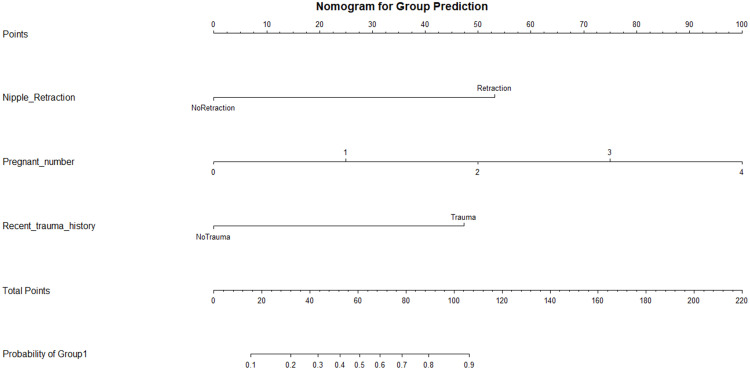
Nomogram model for predicting the risk of PCM.

A logistic regression model was established using R software to create a nomogram for predicting the risk of PCM. The multivariate logistic regression analysis results showed that recent trauma history, nipple retraction and number of pregnancies (P < 0.05) were statistically significant. These three factors were then used to establish the prediction nomogram model for PCM. The nomogram is shown in Fig 3. In the figure, the corresponding points for each variable can be found on the axis for that variable. By drawing a vertical line from that point to the corresponding point on the score scale, the score for each variable can be obtained. The total score is calculated by summing the scores for all variables, and this total score is used to predict the risk of developing PCM.

The scoring system presented in Table 3: Detailed Stratified Scores for Each Factor was developed based on multivariate regression coefficients. The total score is calculated by summing the scores for all variables, and this total score is used to predict the risk of developing PCM.

**Table 3:** Nomogram scoring system.

Predictor	Attribute	Points assigned
Nipple Retraction	Nipple Retraction	53.0
	No Retraction	0
Number of Pregnancies	—continuous—	25.0(per unit increase)
Recent trauma history	Yes	47.4
	No	0

### Evaluation of the prediction model for PCM risk

**Fig 4:**
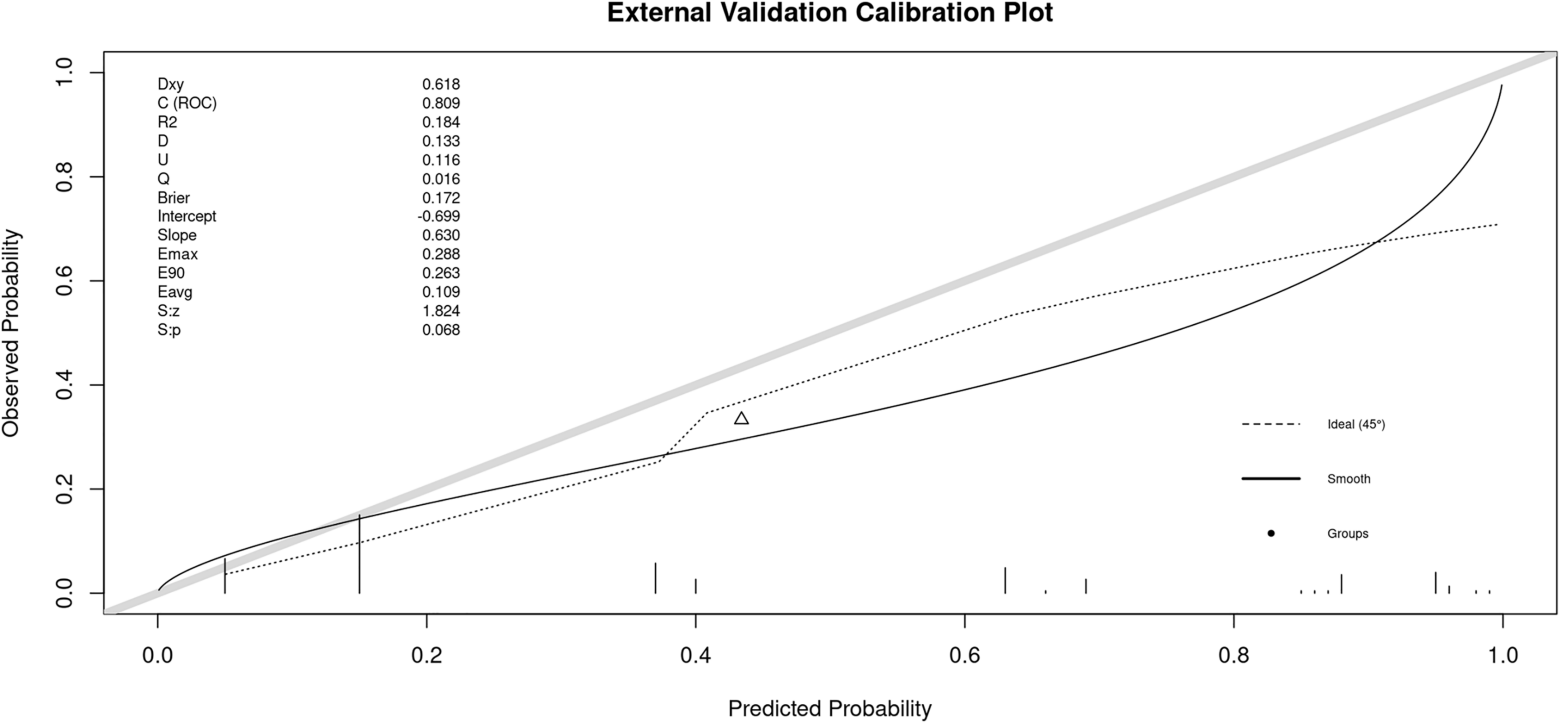
External validation calibration curve for the PCM nomogram model.

The external validation calibration plot for the nomogram model is shown in Fig 4. The x-axis represents predicted PCM risk, and the y-axis represents observed risk. The dashed 45° line indicates perfect calibration, while the solid smooth curve reflects the model’s actual calibration in the external cohort. Overall, the smooth curve follows the ideal line reasonably well at low-to-moderate predicted probabilities, but diverges at higher risk levels, suggesting some miscalibration in the high-probability range. Consistent with this visual pattern, the calibration intercept was −0.699 and the calibration slope was 0.630, indicating that predicted risks are systematically a bit too high and somewhat over-extreme (i.e., mild overfitting) when applied externally. The Brier score was 0.172, with an average absolute calibration error (Eavg) of 0.109 and a maximum error (Emax) of 0.288, supporting acceptable—though not perfect—calibration in external validation. The model’s discrimination in the external cohort remained good (C-index/C(ROC) = 0.809).

The statistical power of the study is sufficient. For key risk factors (nipple retraction, recent trauma history, and number of pregnancies), the current sample size (82 per group) provides >95% power to detect odds ratios (ORs) above 1.5. Although TSH and long-term trauma history show comparatively lower power (≈65–85%), they are not the primary targets and their confidence intervals include the null (e.g., TSH OR = 1.145, 95% CI: 0.70–1.88). In multivariable logistic regression, all variance inflation factors (VIFs) are < 5, indicating no meaningful multicollinearity and supporting model stability. With 82 cases in the case group and five predictors in the model, the events-per-variable (EPV) exceeds 10, suggesting an adequate sample size. In the development set, Cox & Snell R2 = 0.505 indicates strong explanatory capacity; as expected, explained variation decreases in external validation (R2 = 0.184), but remains acceptable given cohort differences.

### ROC curve for the prediction model of PCM risk

**Fig 5:**
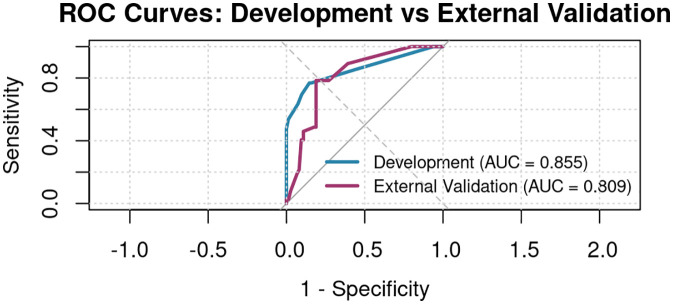
ROC curves for the PCM nomogram model (development AUC = 0.855; external validation AUC = 0.809).

The ROC curves of the PCM nomogram model are shown in Fig 5. In the development cohort, the ROC curve is close to the upper-left corner, yielding an AUC of 0.855, which indicates strong discrimination. In the external validation cohort, the AUC was 0.809, showing a modest decline compared with development but still reflecting good ability to distinguish PCM cases from controls. Overall, these results demonstrate that the nomogram maintains robust predictive performance, with high discriminative accuracy and acceptable generalizability.

## Discussion

PCM is a non-bacterial inflammatory condition that occurs outside of lactation and is considered an autoimmune disease [1,19]. Its incidence has been rising annually. It is characterized by an uncertain etiology, prolonged course, high recurrence rate, and difficulty in treatment [6]. Imaging features often resemble those of breast cancer, which may lead to misdiagnosis [20]. Current treatments for PCM include both surgical and non-surgical options. For patients with different clinical presentations and disease stages, the treatment principle is to reduce recurrence while preserving the aesthetic appearance of the breast. Non-surgical treatments include antibiotics, anti-tuberculosis therapy, corticosteroids, and traditional Chinese medicine [4]. Surgical treatment generally involves segmental resection of the affected area, with complete removal of the lesion to reduce recurrence. In cases of larger resections, adjacent healthy glandular tissue may be transplanted from the same side, followed by local filling and reshaping to maintain the appearance of the breast. The etiology and risk factors of PCM have gradually attracted the attention of clinicians and epidemiologists. Various factors, including smoking, obesity, inverted nipples, and reproductive factors (such as the number of pregnancies and duration of breastfeeding), have been reported to be associated with the disease [21,22]. The nomogram, based on multiple factor regression analysis, integrates several independent risk factors and presents the individual’s risk of a clinical event in a visual format. Medical staff can simply add up the scores of each predictor according to the patient’s situation to estimate the probability of an event occurring.

This study retrospectively analyzed the clinical characteristics of 82 patients with PCM treated in our hospital, conducted univariate and multivariate analysis to identify associated risk factors, established a nomogram prediction model, and validated the model using the ROC curve and calibration curve. Through logistic regression analysis, we identified three independent risk factors for the occurrence of PCM: recent trauma history (OR=11.154, P < 0.001, 95% CI: 2.936–42.382), nipple retraction (OR = 20.128, P < 0.001, 95% CI: 5.952–68.072) and number of pregnancies (OR = 0.343, P < 0.001, 95% CI: 0.189–0.624). A nomogram prediction model based on these three risk factors was developed to predict the individual risk of disease occurrence. Fig 3 presents the intuitive graphical model that integrates multiple independent predictors, providing an individualized risk prediction for PCM.

Fig 3 shows: (1) nipple retraction (score 53.0) increase the weight of the nomogram model’s prediction of PCM risk. Nipple retraction is a well-recognized risk factor. In this study, both univariate χ2 tests and multivariate logistic regression analysis showed significant differences between groups (P < 0.05), which is consistent with published studies. Nipple retraction is a common anatomical abnormality that directly leads to abnormal ductal morphology, causing blockage of the main milk ducts and leading to the accumulation of milk and ductal epithelium damage, triggering local inflammation and further fibrous tissue proliferation, which exacerbates the condition and may form nodules, masses, or abscesses. Women with nipple retraction have a significantly higher risk of PCM, which may be related to abnormal ductal development or inflammation due to pressure from the inverted nipples [23,24]. Therefore, inverted nipples are an important clinical risk factor for PCM. The nomogram model suggests that this factor plays an essential role in risk prediction, further emphasizing the need for timely diagnosis and treatment for patients with this condition. Notably, the high odds ratio observed for nipple retraction may reflect both its clinical relevance and the potential influence of selection bias inherent in retrospective data collection. This association should be further validated in prospective, multicenter cohorts to confirm its generalizability. (2) The risk of developing PCM significantly increases with the number of pregnancies. Specifically, having three pregnancies (score: 74.0 points) substantially enhances the predictive weight of the nomogram model for PCM risk. During pregnancy and lactation, estrogen, progesterone, and prolactin levels significantly increase, all of which can directly act on the breast tissue. Excessive estrogen levels promote continuous proliferation of breast tissue, leading to the reconstruction of breast lobule structures, while elevated prolactin levels cause milk secretion behavior that may lead to ductal expansion, inducing a series of pathological changes and increasing the probability of PCM development. In some research of PCM patients, it was pointed out that postpartum hormonal disorders and abnormal hormone stimulation of ductal epithelium leading to increased secretion, with the possibility of ductal content overflow causing PCM [25,26]. The repeated comprehensive action of various hormones on the breast during multiple pregnancies, causing the breast structure to undergo multiple reconstructions, may be the reason for the increased risk of PCM development. Studies have also indicated that prolactin may be an important pathological factor in the occurrence and recurrence of PCM [27]. (3) Recent trauma history (score 47.4 points) will increase the weight of the nomogram prediction model on the risk of PCM development. This study included breast trauma as a research factor because, in the clinical work of nearly 5 years, it was found that patients with PCM admitted to the breast surgery department of this hospital often mentioned breast trauma when asked about the cause, such as being kicked by their children, collisions with the patient’s breast, or being hit by domestic large pets. Patients usually find breast lumps within 1 week after trauma and develop rapidly. Such experiences also pose a risk of damaging the breast and causing secretions to overflow from the ducts, which is consistent with the mechanism of PCM development. Therefore, this study classified massage and milk expression history as part of breast trauma history and analyzed their role in the course of PCM along with many other factors. In the univariate analysis, breast trauma (including massage and milk expression history) was correlated with the occurrence of PCM. The results of the multivariate regression analysis showed that breast trauma was an independent risk factor for the development of PCM, P < 0.05; Trauma may damage breast ducts, leading to impaired ductal patency and milk overflow into tissue spaces, which may mediate immune inflammatory responses, thus progressing to PCM.

In this study’s univariate analysis, there were significant differences in BMI and blood lipid levels between groups, with P values all less than 0.05. However, after multivariate regression analysis, the differences in BMI and blood lipid levels between groups were not significant and were not independent risk factors for PCM. The reason may be the close relationship between BMI and blood lipid levels, and blood lipid levels or BMI may be positively correlated with the number of pregnancies. Therefore, when controlling for the variable of pregnancy times in the multivariate regression analysis, the intergroup differences in blood lipid levels and BMI would correspondingly lose significance; Long-term trauma history, with a p-value of 0.626, was not statistically significant and is therefore not considered an independent risk factor for PCM. Regarding the relationship between prolactin levels and the occurrence of PCM, some studies have confirmed that elevated prolactin is related to PCM, but this study’s univariate analysis failed to prove the significance of intergroup differences in prolactin, and it is not considered to be related to the occurrence of PCM. The reason may be that the sample size of this study is not large enough. In this study, whether it was univariate or multivariate analysis, smoking was not a risk factor for PCM. The number of smokers included in this study was very few, which may be related to regional cultural customs, and this result may have regional differences. ANCA have received much attention in recent years, playing a certain role in autoimmune diseases such as ulcerative colitis, autoimmune hepatitis, and polyarteritis nodosa. Considering that PCM belongs to autoimmune diseases, this study also included the detection results of ANCA in the analysis, but the univariate analysis showed no significant intergroup differences in ANCA expression.

This study used the ROC curve and calibration curve to internally validate the constructed nomogram prediction model, and the results showed: The ROC curve is close to the upper left corner, indicating good performance of the model. The area under the ROC curve (AUC) was 0.855 (95% CI: 0.800–0.910), close to 1, demonstrating that this model has strong discriminatory ability and high prediction accuracy. In the external validation cohort, the AUC remained good at 0.809, supporting satisfactory generalizability. Using bootstrap resampling, the external calibration curve tracked the 45° ideal line reasonably well, though some deviation was observed at higher predicted risks, suggesting mild overestimation in the upper range. Overall, a nomogram incorporating three key risk factors—recent trauma history, nipple retraction, and number of pregnancies—showed good discrimination and acceptable calibration, enabling individualized prediction of PCM risk.

In Conclusion: The nomogram prediction model, constructed based on three key risk factors for PCM development—recent trauma history, nipple retraction and number of pregnancies—demonstrates strong predictive capability and high accuracy. This model holds significant clinical value in identifying high-risk populations and guiding the implementation of preventive and intervention measures for those at elevated risk [26,27], As an individualized risk prediction tool, this model enhances the ability to forecast PCM onset in patients with relevant risk factors, enabling early intervention and targeted prevention. Moreover, this study not only provides a novel perspective on PCM prevention but also plays a crucial role in raising public awareness and education, ultimately contributing to more effective disease prevention strategies.

According to the results of this study, clinical practice should emphasize vigilance in young and middle-aged female patients with a recent history of trauma, nipple retraction, and a higher number of pregnancies—particularly three or more—to minimize the rate of missed diagnoses and misdiagnoses of PCM. Additionally, follow-up and patient education should be enhanced for women in this demographic who have experienced pregnancy. Patients should be informed about the significance of each risk factor in PCM development, advised to avoid breast trauma, and encouraged to actively correct nipple inversion.

The limitations of this study include: (1) Possible bias due to spontaneous regression of masses in some patients, potentially leading to enrollment of patients with more severe conditions. (2) The relatively small sample size (n = 164), although including all eligible PCM cases during the study period, may reduce the statistical power and restrict the generalizability of the findings. (3) Only internal validation using bootstrapping was performed due to the absence of an external dataset; future studies should externally validate the model with independent, multicenter data to avoid potential overfitting. (4) The study population was limited to Han Chinese women from a single institution, which may restrict the applicability of the model to other ethnic or geographic groups. (5) Several potentially important variables—such as family history, socioeconomic status, and environmental exposures—were not available in the dataset due to the retrospective design. Future prospective studies should aim to include these factors to develop a more comprehensive prediction model.

Future research directions: (1) This study identified recent trauma as a direct contributing factor to PCM development, aligning with previously proposed mechanisms. This opens grounds for future studies and further considerations. Further research is needed to investigate whether trauma-induced damage to ductal epithelial cells and subsequent leakage of ductal contents into tissue spaces may trigger an autoimmune response. Thus, the exact mechanisms of trauma-induced PCM require additional exploration. (2) Conducting multicenter studies with larger sample sizes is essential for minimizing selection bias and enhancing the credibility and generalizability of research findings.

## Supporting information

S1 File English version of the ethical approval document. (DOCX)

S2 File Original Chinese ethical approval document. (PDF)

S3 File Human participants research checklist. (DOCX)
