# Supplementary material for: Risk factor analysis and development of a nomogram prediction model for Plasma Cell Mastitis
Source: PLoS One. 2025 Dec 9;20(12):e0338711. doi: 10.1371/journal.pone.0338711 (PMC12688106; doi:10.1371/journal.pone.0338711)
Supplement: S1 File — (DOCX) [file pone.0338711.s001.docx]

Hangzhou Women’s Hospital (Hangzhou Maternal and Child Health Care Hospital)

Hangzhou First People's Hospital Qianjiang New City Branch

Ethics Project Approval Form

[2022] Medical Ethics Review K No. (6) - 03

| Event/Project Name: **Risk Factor Analysis and Development of a Nomogram Prediction Model for Plasma Cell Mastitis** | | | | |
| --- | --- | --- | --- | --- |
| Reporting Department: Breast Surgery Department | Applicant: Ma Xiaowen | | Application Matter: Writing a Thesis | |
| Application Matter: Writing a Thesis | Review Date: 2022.6.9 | | Meeting Location: / | |
| Ethics Committee Contact: Huang Jian | | | Contact Number: 0571-56005074 | |
| Review Materials: Ethics Review Application Form, Ethics Review Worksheet, Research Protocol, Scientific Research Project Confidentiality Commitment, Principal Investigator's Resume, Other Materials: | | | | |
| Voting Results: Three members of the Ethics Committee carefully reviewed and discussed the aforementioned documents and voted. The number of voters: 3 people, results are as follows: | | | | |
| Agree | Agree with Necessary Revisions | Re-review after Necessary Revisions | Terminate or Suspend Approved Trials | Disagree |
| (3) votes | (0) votes | (0) votes | (0) votes | (0) votes |
| Review Comments:  After review by the hospital's Ethics Committee, the research project meets ethical requirements and is approved to proceed to the next stage of research work.  Hangzhou Women’s Hospital (Hangzhou Maternal and Child Health Care Hospital)  Ethics Committee Seal  Chairman's Signature:  Date: June 9, 2022 | | | | |
