# Supplementary material for: Risk factor analysis and development of a nomogram prediction model for Plasma Cell Mastitis
Source: PLoS One. 2025 Dec 9;20(12):e0338711. doi: 10.1371/journal.pone.0338711 (PMC12688106; doi:10.1371/journal.pone.0338711)
Supplement: S2 File — (PDF) [file pone.0338711.s002.pdf]

杭州市妇产科医院（杭州市妇幼保健院）
杭州市第一人民医院钱江新城院区

伦理项目审批表

【2022】医伦审 K 第（6）号-03

事件/项目名称：浆细胞性乳腺炎的危险因素分析及预测模型建立

报审科室：乳腺科	申请人：马啸文	申请事项：撰写论文
审查方式： 快审审查	审查时间：2022.6.9	会议地点：/

伦理委员会联系人：黄坚	联系电话： 0571-56005074

审查材料：伦理审查申请表、伦理审查工作表、研究方案、科研项目保密承诺书、主要研究者履历、其他资料；

投票结果：伦理委员会 3 位成员对上述文件进行了认真的审查和讨论，并进行了投票表决，其中投票人数： 3 人，结果如下：

同意	作必要修正后同意	作必要修正后重审	终止或暂停已批准试验	不同意
(3)票	(0)票	(0)票	(0)票	(0)票

审查意见：

经本院伦理委员会审查，该研究项目符合伦理要求，同意其进行下一步的研究工作。

杭州市妇产科医院（杭州市妇幼保健院）
伦理委员会（盖章）

杭州市妇产科医院 伦理委员会

主任委员签名：

日期 ：2022 年 6 月 9 日

F201008

2016-06-A
